# Preparation of Biodegradable Oligo(lactide)s-Grafted Dextran Nanogels for Efficient Drug Delivery by Controlling Intracellular Traffic

**DOI:** 10.3390/ijms19061606

**Published:** 2018-05-30

**Authors:** Yuichi Ohya, Akihiro Takahashi, Akinori Kuzuya

**Affiliations:** 1 Department of Chemistry and Materials Engineering, Faculty of Chemistry, Materials and Bioengineering, Kansai University, 3-3-35 Yamate, Suita, Osaka 564-8680, Japan; kuzuya@kansai-u.ac.jp; 2 Organization for Research and Development of Innovative Science and Technology (ORDIST), Kansai University, 3-3-35 Yamate, Suita, Osaka 564-8680, Japan; akki.taka4@gmail.com

**Keywords:** biodegradable nanogel, disulfide bond, drug delivery, cellular traffic

## Abstract

Nanogels, nanometer-sized hydrogel particles, have great potential as drug delivery carriers. To achieve effective drug delivery to the active sites in a cell, control of intracellular traffic is important. In this study, we prepared nanogels composed of dextran with oligolactide (OLA) chains attached via disulfide bonds (Dex-*g*-SS-OLA) that collapse under the reductive conditions of the cytosol to achieve efficient drug delivery. In addition, we introduced galactose (Gal) residues on the nanogels, to enhance cellular uptake by receptor-mediated endocytosis, and secondary oligo-amine (tetraethylenepentamine) groups, to aid in escape from endosomes via proton sponge effects. The obtained Dex-*g*-SS-OLA with attached Gal residues and tetraethylenepentamine (EI_4_) groups, EI_4_/Gal-Dex-*g*-SS-OLA, formed a nanogel with a hydrodynamic diameter of ca. 203 nm in phosphate-buffered solution. The collapse of the EI_4_/Gal-Dex-*g*-SS-OLA nanogels under reductive conditions was confirmed by a decrease in the hydrodynamic diameter in the presence of reductive agents. The specific uptake of the hydrogels into HepG2 cells and their intercellular behavior were investigated by flow cytometry and confocal laser scanning microscopy using fluorescence dye-labeled nanogels. Escape from the endosome and subsequent collapse in the cytosol of the EI_4_/Gal-Dex-*g*-SS-OLA were observed. These biodegradable nanogels that collapse under reductive conditions in the cytosol should have great potential as efficient drug carriers in, for example, cancer chemotherapy.

## 1. Introduction

Nanogels are nanometer-sized hydrogel particles (diameter <1 µm) composed of three-dimensional networks of physically (or chemically) cross-linked polymer chains. Such nanogels have attracted much attention over the last decade due to their potential for applications in biomedical fields, such as for drug delivery systems (DDS) and bioimaging [1,2,3,4,5,6,7,8,9,10,11,12,13]. A physically cross-linked nanogel was first reported by Akiyoshi and coworkers in 1997 [14]. They reported that hydrophobized polysaccharides can form physically cross-linked nanogels in aqueous solution and entrap both hydrophobic and hydrophilic molecules, such as proteins [4]. Physical entrapment and the release behavior of proteins into and from nanogels have been investigated for applications as DDS carriers [9]. Biodegradability is one of the crucial factors for appropriate DDS carriers. Biodegradation offers several advantages, such as facilitation in releasing encapsulated molecules and excretion of the vehicles after release of the bioactive agents. We also reported biodegradable dextran-*graft*-oligo(l-lactide) (Dex-*g*-OLLA) nanogels and their stabilization by mixing with dextran-*graft*-oligo(d-lactide) (Dex-*g*-ODLA) via stereocomplex formation [15]. We succeeded in preparing protein-loaded Dex-*g*-OLLA nanogels using lysozyme as a model protein and found that the lysozyme-loaded nanogels showed sustained release of lysozyme for one week without denaturation in phosphate-buffered saline (PBS) at 37 °C [16].

**Figure 1:**
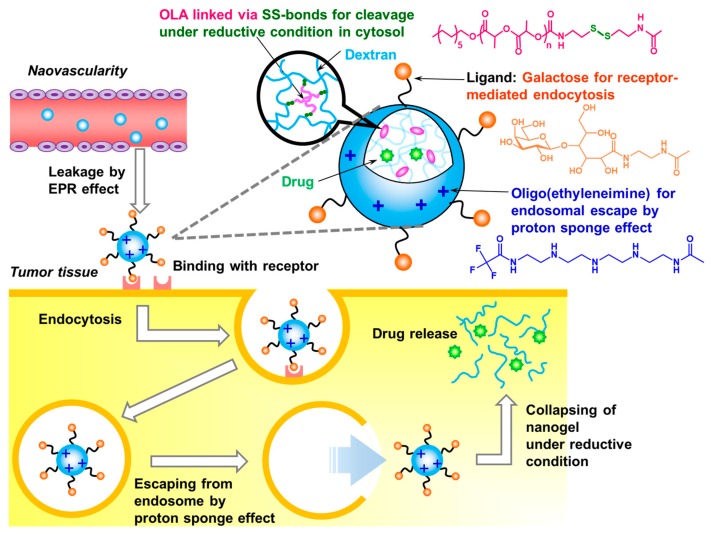
Schematic illustration for the concept of this work and the design of nanogel. EPR: enhanced permeability and retention, OLA: oligolactide, SS: disulfide.

To apply drug carriers in cancer chemotherapy, some additional functionalities are desired (Figure 1). First, enhanced permeability and retention (EPR) effects are necessary to accumulate the drug carriers in the highly vascular solid tumor tissue by passive targeting. Macromolecules with large hydrodynamic diameters or nanoparticles are more likely to be leaked from the neovascular tissue to accumulate in tumor tissue and are retained in the tissues due to the lack of lymph nodes [17,18]. Second, the carrier uptake into the cancer cells should occur via specific or non-specific interactions [19,20,21]. Endocytosis is one of the well-known cellular uptake processes, which only proceeds after the specific binding of ligands or antibodies against receptors or epitopes located on the cell membrane [21,22,23]. Third, after uptake into cancer cells, the carriers should escape from the endosomes into the cytosol. The proton sponge effect via protonation of secondary amine groups (like polyethyleneimine) on the carriers is one promising strategy for endosomal escape [24,25,26]. Finally, the carriers should collapse or be dissociated to release the drugs. Because the cytosol generally possesses reductive conditions, disulfide bonds have been utilized to release drugs in the cytosol, either by dissociation of the nanoparticles or by cleavage of the degradable bonds between carriers and drugs to expose the drugs to active sites or organelles in the target cells [27,28,29].

In this study, to achieve highly efficient cellular drug delivery to the cytosol by controlling intercellular traffic, we prepared nanogels composed of dextran (Dex) with oligolactide (OLA) chains attached via disulfide bonds to be collapsed under the reductive conditions of cytosol (Figure 1). In addition, the nanogels were equipped to enhance cellular uptake and escape from endosomes. The galactose (Gal) residue was chosen as a model ligand to enhance uptake into liver-derived cancer cells by receptor-mediated endocytosis [30,31]. Additionally, oligo-amine (tetraethylenepentamine, EI_4_) groups were attached to the nanogels to enhance escape from endosomes by proton sponge effects [24]. Finally, we investigated the interaction of the obtained nanogels with cells as well as their intracellular behavior.

## 2. Results and Discussion

### 2.1. Synthesis of Graft Copolymer

**Scheme 1:**
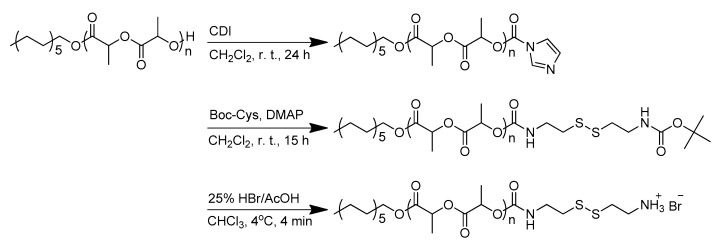
Synthesis of aminoethyl-disulfanyl-ethyl-OLA (hydrogen bromide salt) (OLA-SS-NH_3_^+^Br^−^). CDI: *N*,*N*’-carbonyldiimidazole, DMAP: 4-(*N*,*N*-dimethylamino)-pyridine.

**Scheme 2:**
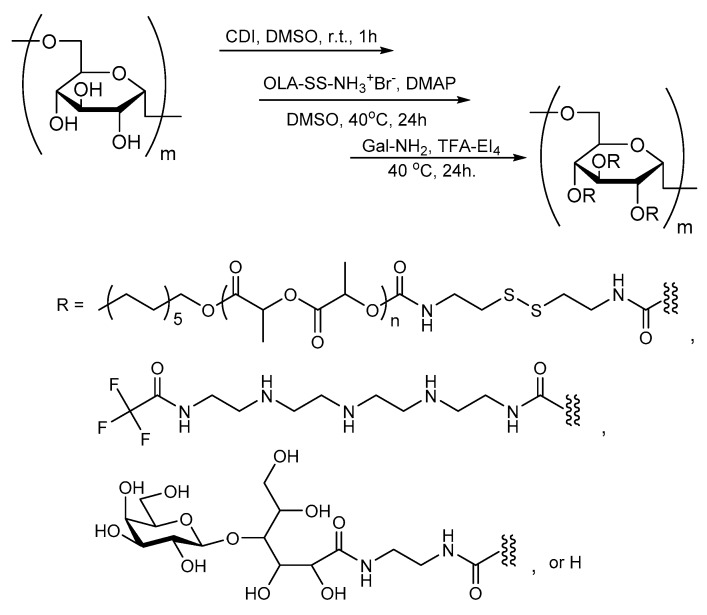
Synthesis of tetraethylenepentamine/galactose-modified dextran-*graft*-disulfanyl-ethyl-OLA (EI_4_/Gal-Dex-*g*-SS-OLA). DMSO: dimethylsulfoxide, TFA: trifluoroacetic acid.

OLA having a terminal primary amine group via a disulfide bond spacer (aminoethyl-disulfanyl-ethyl-OLA, OLA-SS-NH_2_) was synthesized according to Scheme 1 (Supporting Information, Figure S1). Tetraethylenepentamine/galactose-modified dextran-*graft-*disulfanyl-ethyl-OLA (EI_4_/Gal-Dex-*g*-SS-OLA) was prepared according to Scheme 2. Other control copolymers were also prepared by the variation of introduced components. The characterization of EI_4_/Gal-Dex-*g*-SS-OLA was carried out by ^1^H nuclear magnetic resonance (NMR) spectroscopy (Figure S1), confirming the existence of Dex (3.05–3.90 and 4.83 ppm), OLA-SS units (1.08, 2.50, and 3.94 ppm), ethylene groups of EI_4_ (2.30–2.60 ppm), and Gal residues (2.67–2.80, 3.05–3.90, and 4.38 ppm). The amounts of OLA-SS units, EI_4_ groups, and Gal residues were calculated, by the integral ratios of the ^1^H NMR spectrum, to be 2.5, 35, and 30 molecules per Dex molecule, respectively. These values correspond to degrees of introduction of 0.9, 13.2, and 11.3 mol % per sugar unit of Dex, respectively.

### 2.2. Characterization of Nanogels and Dissociation under Reductive Conditions

Nanogels were prepared by a dialysis method. The hydrodynamic diameters of the nanogels were investigated with dynamic light scattering (DLS) measurements (Figure S3). The Z-averages of the hydrodynamic diameters (*D*_h_) of Dex-*g*-OLA, Dex-*g*-SS-OLA, and EI_4_/Gal-Dex-*g*-SS-OLA nanogels are estimated to be 141, 141, and 203 nm, respectively, and their top peak contributions are 199, 213, and 263 nm. Dex-*g*-OLA and Dex-*g*-SS-OLA nanogels show similar *D*_h_, however the EI_4_/Gal-Dex-*g*-SS-OLA nanogel shows a slightly larger *D*_h_ value, suggesting electrostatic repulsion and higher hydration of EI_4_ groups. The stability of the nanogel against dilution was evaluated through the critical aggregation concentration (CAC) value by a fluorescence probe method, using pyrene as the fluorescence probe (Figure S4). The CAC value of Dex-*g*-SS-OLA nanogels is estimated to be 5.9 × 10^−4^ wt %. This result is similar to the case of Dex-*g*-OLA nanogels previously reported [15].

**Figure 2:**
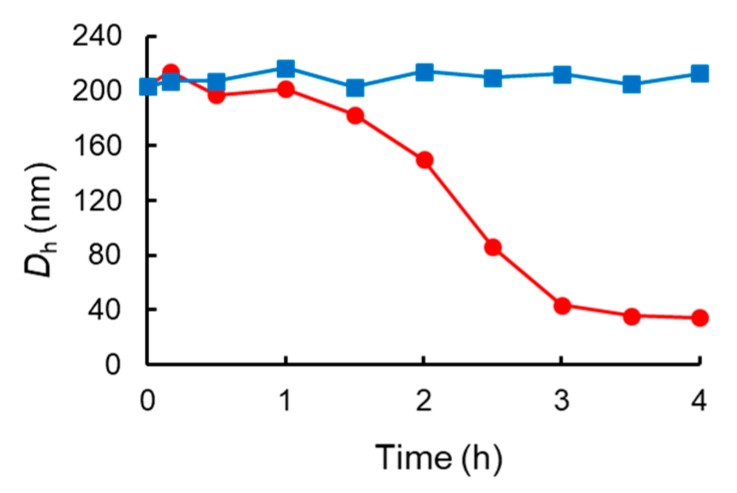
Time course of the Z-average of the hydrodynamic diameter (*D*_h_) for the EI_4_/Gal-Dex-*g*-SS-OLA nanogel monitored by dynamic light scattering (DLS) in the presence (red circles) or absence (blue squares) of 10 mM glutathione (GSH) in phosphate buffer (PB) solution.

The stability and dissociation behavior of the nanogels under reductive conditions were investigated by monitoring *D*_h_ after the addition of a reductive agent. Figure 2 shows the time course of the average *D*_h_ for the EI_4_/Gal-Dex-*g*-SS-OLA nanogel monitored by DLS in the presence or absence of 10 mM glutathione (GSH), as a reductive agent. Without GSH, the diameter of the EI_4_/Gal-Dex-*g*-SS-OLA nanogel does not change. On the other hand, the hydrodynamic diameter decreases in the presence of GSH to be less than 10 nm in 1 h. These results suggest that the disulfide bonds are cleaved in the presence of GSH as a reductive reagent, and the EI_4_/Gal-Dex-*g*-SS-OLA nanogels collapse. 

**Figure 3:**
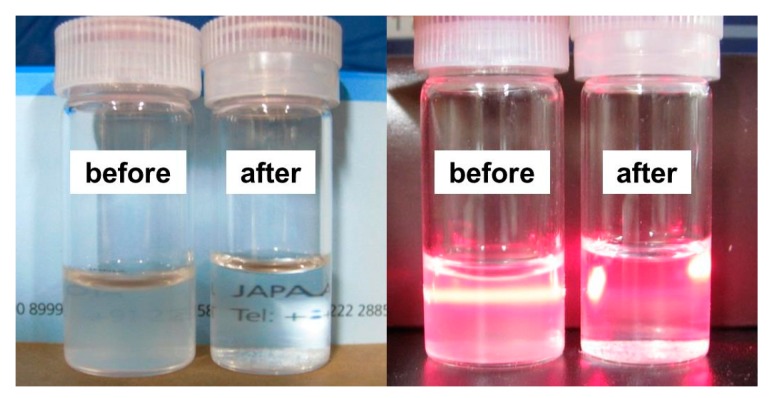
Photographs of EI_4_/Gal-Dex-*g*-SS-OLA nanogel before and after 24 h incubation in the presence of 10 mM dithiothreitol (DTT) in PB solution at 25 °C with (**right**) and without (**left**) laser beam irradiation.

**Figure 4:**
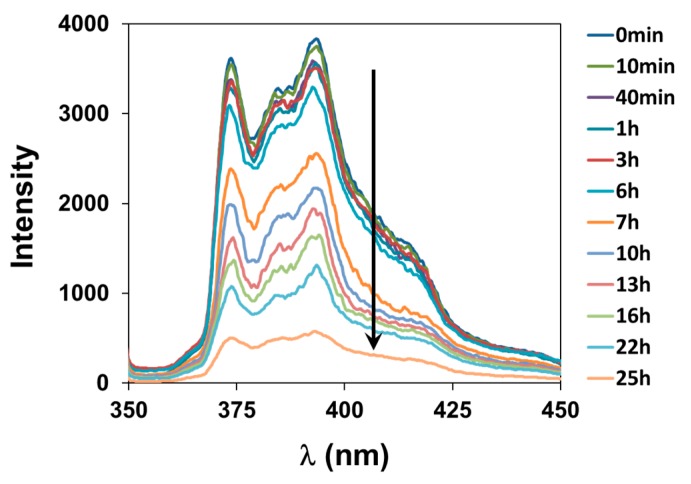
Time course of fluorescence spectra of pyrene entrapped in EI_4_/Gal-Dex-*g*-SS-OLA nanogels after addition of 10 mM DTT in PB solution at 25 °C. Black arrow means time course.

The dissociation behavior of the EI_4_/Gal-Dex-*g*-SS-OLA nanogels was investigated by other techniques. Figure 3 shows photographs of the original EI_4_/Gal-Dex-*g*-SS-OLA nanogel solution and the nanogel solution after 24 h incubation in the presence of 10 mM of dithiothreitol (DTT) as a reductive agent. The nanogel solution before incubation was slightly turbid and showed strong scattering by red laser beam irradiation, indicating dispersion of the particles at the hundreds-of-nanometers scale. On the other hand, the nanogel solution after 24 h incubation with DTT was a clear solution and almost no scattering was observed. These results indicate that the aggregates (nanogels) collapse into very small particles or soluble molecules without scattering. Furthermore, the time course of the fluorescence spectra for the EI_4_/Gal-Dex-*g*-SS-OLA nanogels entrapping pyrene in the presence of 10 mM DTT as a reductive agent is shown in Figure 4. After the addition of DTT, the fluorescence intensity of pyrene gradually decreases. These results indicate that the hydrophobic pyrene molecules are released from the nanogels and precipitate upon their collapse. The change in fluorescence spectra have a different tendency compared to the DLS measurement (Figure 2); the fluorescence decay took nearly 24 h, while the *D*_h_ change in DLS measurements was complete after several hours. The DLS measurements reflect the direct dissociation of a larger nanogel into a partially collapsed nanogel having smaller (several tens of nanometers) diameter. However, the partially collapsed nanogels still have hydrophobic domains which can entrap pyrene molecules. This may be the reason that the complete release of pyrene took a longer time. From all of these results, EI_4_/Gal-Dex-*g*-SS-OLA nanogels are presumably collapsed under the reductive conditions of the cytosol.

**Figure 5:**
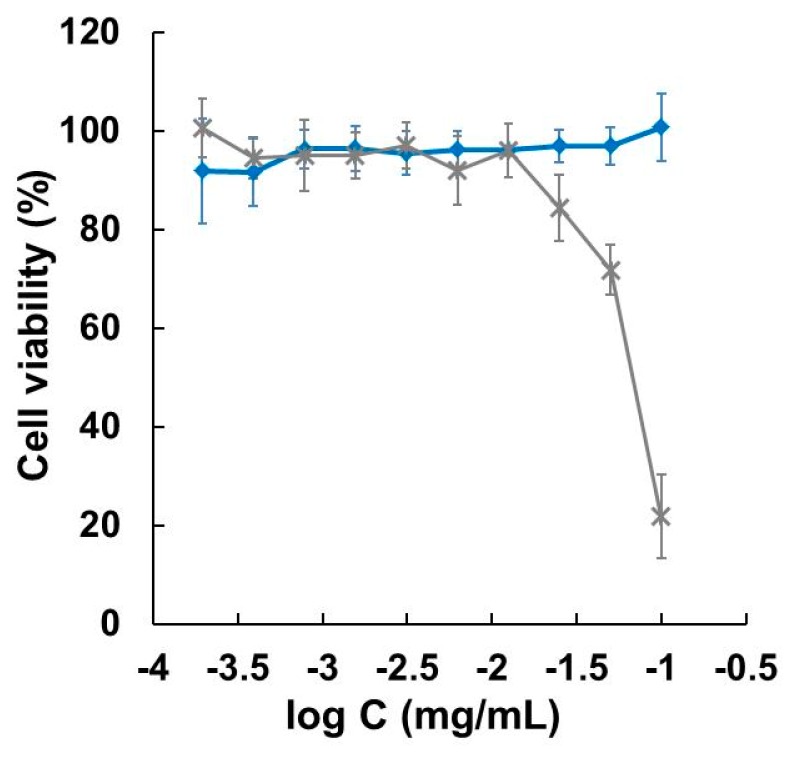
Viability of L929 cells after 24 h incubation as a function of concentrations of (♦) EI_4_/Gal-Dex-*g*-SS-OLA nanogels and (**×**) SDS.

The cytotoxicity of EI_4_/Gal-Dex-*g*-SS-OLA nanogels was investigated against L929 mouse fibroblast cells by the WST-8 assay (Figure 5). Sodium dodecyl sulfate (SDS, surfactant) was used as a positive control. No decrease in cell viability was observed for EI_4_/Gal-Dex-*g*-SS-OLA nanogels below 1.0 × 10^−1^ mg/mL.

### 2.3. Cellular Uptake Behavior of EI_4_/Gal-Dex-g-SS-OLA Nanogels

**Figure 6:**
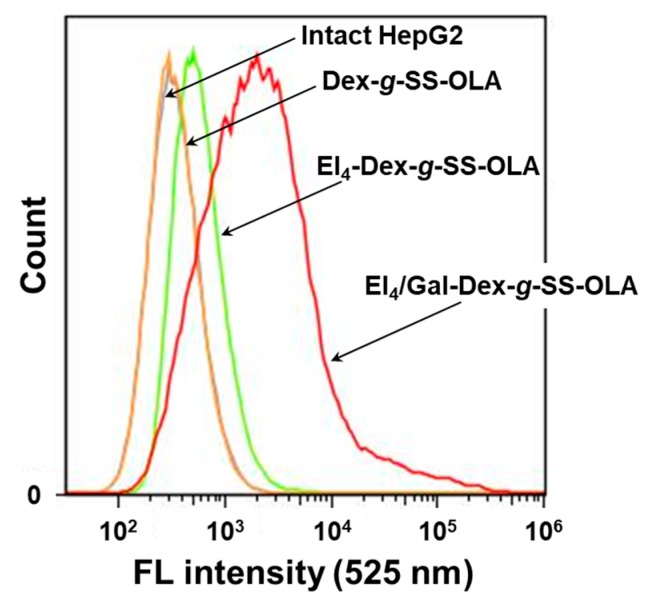
Results of flow cytometric analysis for HepG2 cells incubated with fluorescein isothiocyanate isomer I (FITC)-labeled nanogels for 4 h. Intact HepG2 cells (grey, almost overlapped with orange), Dex-*g*-SS-OLA (orange), EI_4_-Dex-*g*-SS-OLA (light green), EI_4_/Gal-Dex-*g*-SS-OLA nanogels (red). FL: fluorescence.

To investigate the cellular uptake of the nanogels into HepG2 cells, flow cytometric analysis was carried out using fluorescein isothiocyanate isomer I (FITC)-labeled nanogels (Figure 6). A shift to the right (stronger fluorescence intensity at 525 nm) in the cell distribution means that an uptake of FITC-labeled nanogels into HepG2 cells has occurred. After incubation with EI_4_/Gal-Dex-*g*-SS-OLA nanogels, a peak shift to the right is indeed observed compared with intact HepG2 and that incubated with Dex-*g*-SS-OLA nanogels without galactose residues. HepG2 cells incubated with EI_4_-Dex-*g*-SS-OLA nanogels with cationic EI_4_ groups but without galactose residues show a slight shift to the right, suggesting an electrostatic interaction of cationic groups of EI_4_-Dex-*g*-SS-OLA nanogels with HeG2 cells. Since EI_4_/Gal-Dex-*g*-SS-OLA nanogels induce a larger shift to higher fluorescence intensity compared with EI_4_-Dex-*g*-SS-OLA nanogels, galactose receptor-mediated specific binding of EI_4_/Gal-Dex-*g*-SS-OLA nanogels against HepG2 cells is suggested.

**Figure 7:**
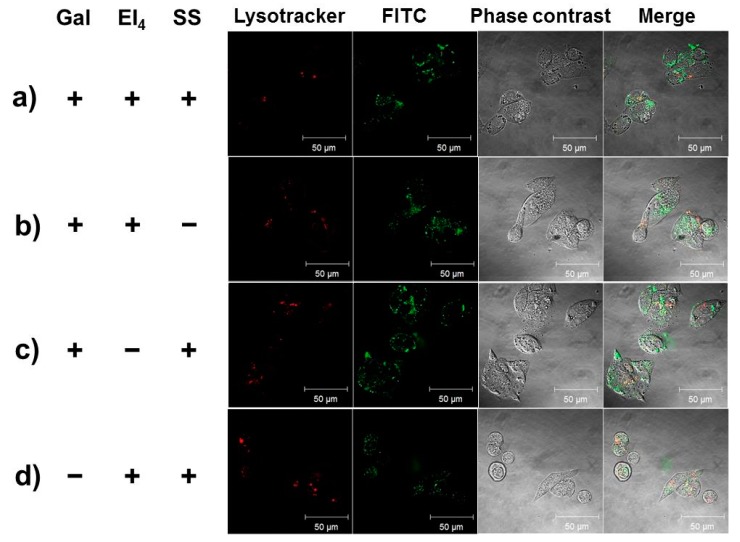
Confocal laser scanning microscopy (CLSM) images of HepG2 cells with various FITC-labeled nanogels and LysoTracker RED after 5 min incubation at 37 °C. (**a**) EI_4_/Gal-Dex-*g*-SS-OLA, (**b**) EI_4_/Gal-Dex-*g*-OLA, (**c**) Gal-Dex-*g*-SS-OLA, and (**d**) EI_4_-Dex-*g*-SS-OLA. Scale bar: 50 µm.

**Figure 8:**
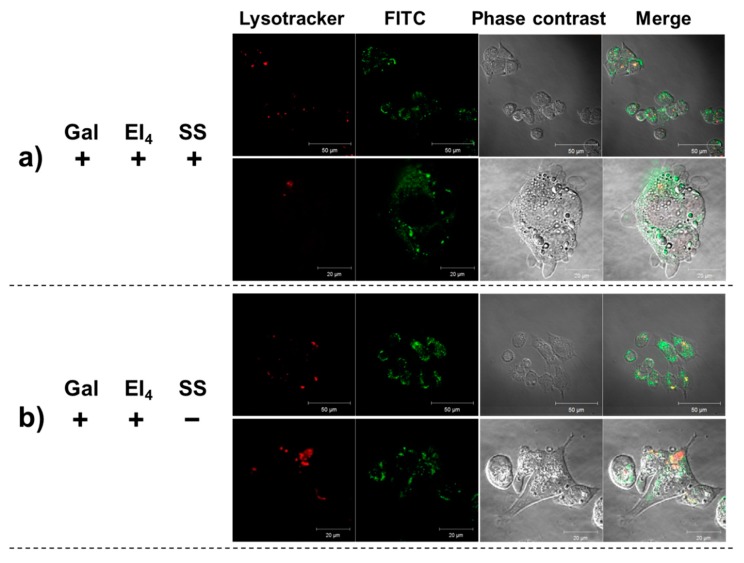

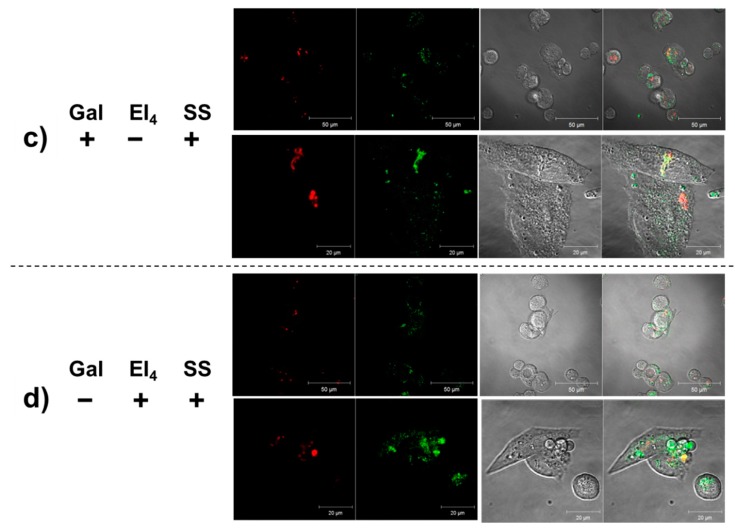
CLSM images of HepG2 cells with various FITC-labeled nanogels and LysoTracker RED after 5 h incubation at 37 °C. (**a**) EI_4_/Gal-Dex-*g*-SS-OLA, (**b**) EI_4_/Gal-Dex-*g*-OLA, (**c**) Gal-Dex-*g*-SS-OLA, and (**d**) EI_4_-Dex-*g*-SS-OLA. Scale bar: 50 µm (top row of each panel) and 20 µm (bottom row of each panel).

We performed confocal laser scanning microscopy (CLSM) of the HepG2 cells incubated with the nanogels to confirm locations of the nanogels in each cell and their collapse. The cells were incubated with FITC-labeled nanogels, EI_4_/Gal-Dex-*g*-SS-OLA, EI_4_/Gal-Dex-*g*-OLA (without –SS– bonds), Gal-Dex-*g*-SS-OLA (without EI_4_), and EI_4_-Dex-*g*-SS-OLA (without Gal). The results are shown in Figure 7 and Figure 8. The nanogels and lysosomes are observed as green fluorescence by FITC and red fluorescence by LysoTracker RED, respectively. Figure 7 shows CLSM images of HepG2 cells with various nanogels after 5 min incubation at 37 °C. EI_4_/Gal-Dex-*g*-SS-OLA, Gal-Dex-*g*-SS-OLA, and EI_4_/Gal-Dex-*g*-OLA nanogels with Gal residues show relatively strong green fluorescence at the periphery of the cells, although EI_4_-Dex-*g*-SS-OLA do not show very strong fluorescence. These results suggest that nanogels with Gal residues bind to the HepG2 cells by specific galactose receptor mediated interactions, and the electrostatic interaction between cationic EI_4_ groups on the nanogels and the cells is not dominant. Overlap of red fluorescence of LysoTracker RED and green fluorescence of FITC is not so obvious for all nanogels. This may be because endocytosis is not completed in 5 min, and the observed nanogels exist on the cell surfaces. The cells were further incubated at 37 °C for 5 h (Figure 8). In Figure 8c, the green and red fluorescence spots are observed at almost the same positions for Gal-Dex-*g*-SS-OLA nanogels without EI_4_ groups, suggesting that the Gal-Dex-*g*-SS-OLA nanogels uptaken by receptor-mediated endocytosis were localized at endosomes (or lysosomes). In Figure 8b, the green and red fluorescence spots are observed at different positions for EI_4_/Gal-Dex-*g*-OLA nanogels without SS linkages, suggesting that the EI_4_/Gal-Dex-*g*-OLA nanogels can escape from the lysosome. However, the green fluorescence is observed as spots suggesting the nanogels retain their particle shapes and do not diffuse very much in the cytosol. On the other hand, in Figure 8a, the green fluorescence is spread over a wider area in the cytosol for EI_4_/Gal-Dex-*g*-SS-OLA nanogels. These results suggest that the EI_4_/Gal-Dex-*g*-SS-OLA nanogels collapse and the FITC-labeled copolymer can diffuse in the cytosol. Therefore, the introduction of EI_4_ as a cationic group is effective for escape from the endosomes, and the –SS– group can be cleaved under the reductive conditions of the cytosol to collapse EI_4_/Gal-Dex-*g*-SS-OLA nanogels.

## 3. Materials and Methods

### 3.1. Materials

l-Lactide was purchased from Musashino Chemical Laboratory, Ltd. (Tokyo, Japan) and used without further treatment. Dextran (Dex) (*M*_W_ = 43,000), fluorescein isothiocyanate isomer I (FITC) and ethylenediamine were purchased from Sigma-Aldrich (St. Louis, MO, USA). *N*,*N*’-Carbonyldiimidazole (CDI), 4-(*N*,*N*-dimethylamino)-pyridine (DMAP), di-*tert*-butyl dicarbonate, cystamine dihydrochloride, dithiothreitol (DTT), and lactose were purchased from Wako Pure Chemical Ind., Ltd. (Osaka, Japan). Ethyl trifluoroacetate, tetraethylenepentamine (EI_4_), and glutathione (GSH) were purchased from Tokyo Chemical Industry Co., Ltd. (Tokyo, Japan). LysoTracker RED was obtained from Thermo Fisher Scientific K.K. (Tokyo, Japan). Other reagents were commercial grade and used without further purification. Oligo(l-lactide) (OLA) (Mw = 3070 Da, degree of polymerization = 20) was prepared by the usual ring-opening polymerization of l-lactide using tin(2-ethylhexanoate) (Sn(Oct)_2_) as a catalyst and 1-dodecanol as an initiator, according to the literature [32]. *N*-Lactonoyl-1,4-ethylenediamine (Gal-NH_2_) and mono-*t*-butoxycarbonyl-cystamine (Boc-cystamine) were prepared by the methods reported previously with minor modification [33,34]. *N*-[2-[[2-[[2-[(2-Aminoethyl)amino]ethyl]amino]ethyl]amino]ethyl]-2,2,2-trifluoroacetamide (TFA-EI_4_) was synthesized from ethyl trifluoroacetate and EI_4_ by a previously reported method [35]. Fetal calf serum (FCS) was obtained from Thermo Fisher Scientific (Waltham, MA, USA). Eagle’s minimum essential medium (E-MEM) and Dulbecco’s minimum essential medium (D-MEM) were purchased from Nissui Pharmaceutical Co. (Tokyo, Japan).

### 3.2. Measurements

^1^H NMR spectra were recorded on a JNM-GSX-400 (JEOL, Tokyo, Japan, 400 MHz) nuclear magnetic resonance spectrometer using deuterated solvent. The chemical shifts were calibrated against tetramethylsilane (TMS) and/or solvent signal. The number average molecular weights (*M*n) and polydispersity indices (*M*_W_/*M*n) of OLA were determined by size exclusion chromatography (SEC) (column: TSKgel Multipore HXLM × 2; detector: RI). The measurements were performed using dimethylformamide (DMF) as an eluent at a flow rate of 1.0 mL/min at 40 °C and a series of polystyrenes were used as standards. Hydrodynamic diameters of nanogels were measured by DLS (Zetasizer nano Z ZEN 2600, Malvern Instruments, Ltd., Malvern, UK). Fluorescence emission spectra of pyrene in the presence of nanogels were recorded by a fluorescence spectrophotometer (Shimadzu UV-2400PC, SIMADZU Co., Kyoto, Japan) using the excitation wavelength of 335 nm at room temperature (rt) in PB (phosphate buffer) solution (50 mM, pH 7.4). The critical aggregation concentration (CAC) of the nanogels in PB solution were determined using pyrene as a fluorescence probe. Fluorescence intensities of *I*_1_ and *I*_3_ were measured at the wavelengths of 373 and 383 nm, corresponding to the first and third vibronic bands. The CAC can be obtained from the flexion point in the *I*_1_/*I*_3_ ratio vs. concentration plots.

### 3.3. Synthesis of Aminoethyl-Disulfanyl-Ethyl-OLA (OLA-SS-NH_2_)

Aminoethyl-disulfanyl-ethyl-OLA (OLA-SS-NH_2_) was synthesized according to Scheme 1. OLA (1.00 g, 326 µmol) was dissolved in 3 mL of anhydrous dichloromethane with CDI (159 mg, 977 µmol), and stirred at rt for 24 h. The reaction mixture was poured into ethanol/*n*-hexane (3/7, *v*/*v*) and washed three times. The precipitate was dried in vacuo at rt to give a white powder of activated OLA (CI-OLA) (993 mg, yield 96%). The CI-OLA (993 mg, 314 µmol), Boc-cystamine (152 mg, 942 µmol), and DMAP (25 mg, 314 µmol) were dissolved in anhydrous dichloromethane, and stirred at rt for 15 h. The obtained product was precipitated by ethanol/*n*-hexane (3/7, *v*/*v*), washed three times, and dried in vacuo at rt to give a white powder of Boc-cystamine-OLA (921 mg, yield 87%). Boc-cystamine-OLA (921 mg, 275 µmol) was dissolved in 3 mL of chloroform while cooling at 0 °C. HBr/AcOH (25%) (343 µL, 1.38 mmol) was added to remove the Boc group and the mixture was stirred at 0 °C for 4 min. The reaction mixture was poured into diethylether to precipitate, washed three times, and dried in vacuum at rt to give a white powder of aminoethyl-disulfanyl-ethyl-OLA (OLA-SS-NH_2_) (884 mg, yield 98%).

^1^H NMR (400 MHz, CDCl_3_): δ = 0.89 (t, CH_3_CH_2_–), 1.20–1.38 [m, 20H, CH_3_(CH_2_)_10_CH_2_–], 1.46–1.71 (m, –CHCH_3_–), 2.76–3.36 (br, 4H, –SC*H*_2_CH_2_–), 3.36–3.69 [br, 4H, –OC(=O)NHCH_2_CH_2_S– and –CH_2_NH_2_], 4.06–4.21 (m, –CH_2_CH_2_OCO–), 5.08–5.32 [m, –COCH(CH_3_)O–], 7.91–8.15 (br, –CH_2_NH_2_) (Figure S1).

### 3.4. Synthesis of Tetraethylenepentamine/Galactose-Modified Dextran-Grafted-Disulfanyl-Ethyl-OLA (EI_4_/Gal-Dex-g-SS-OLA)

The synthesis of tetraethylenepentamine/galactose-modified dextran-grafted-disulfanyl-ethyl-OLA (EI_4_/Gal-Dex-*g*-SS-OLA) was carried out according to Scheme 2. Dex (200 mg, 4.65 µmol, 1.23 mmol of glucose unit) was dissolved in 3 mL of anhydrous dimethylsulfoxide (DMSO). CDI (120 mg, 558 µmol) was added, and the solution was stirred at rt for 1 h to activate the hydroxyl groups. The reaction mixture was poured into acetone and washed three times. The precipitate was dried in vacuo at rt to give a white powder (140 mg, yield 55%). The activated Dex (Dex-CI) (140 mg, 2.81 µmol, 110 imidazole groups per macromolecule), OLA-SS-NH_2_ (96 mg, 30.9 µmol), and DMAP (11 mg, 92.7 µmol) were dissolved in 3 mL of anhydrous DMSO, and stirred at 40 °C for 24 h. Gal-NH_2_ (45 mg, 112 µmol) and TFA-EI_4_ (70 mg, 245 µmol) were added and the reaction mixture was stirred at 40 °C for a further 48 h. The reaction mixture was poured into acetone and washed three times. The precipitate was dried in vacuo at rt to give a white powder of EI_4_/Gal-Dex-*g*-SS-OLA (182 mg, yield 99% (based on Dex)). Each component was characterized by ^1^H NMR after hydrolysis using NaOD/D_2_O (Figure S2).

^1^H NMR (400 MHz, NaOD/D_2_O): δ = 1.16 (d, CH_3_CH–), 2.27–2.79 (br, –NHCH_2_CH_2_–), 3.06–3.90 (m, H^2^–H^6^ of Dex and Gal), 3.94 (q, CH_3_CH–), 4.32–4.43 (m, H^1^ of Gal), 4.71–5.13 (br, H^1^ of Dex).

### 3.5. Preparation of Nanogel

EI_4_/Gal-Dex-*g*-SS-OLA (20 mg), dissolved in 2 mL of DMSO, was mixed with 8 mL of water with vigorous stirring at rt. The resulting solution was placed in a dialysis tube (molecular weight cut off (MWCO): 3500), and dialyzed for 24 h against water at rt to remove organic solvent. Next, the aqueous solution was lyophilized to give a white powder of the nanogel (17 mg, yield 88%). To prepare the nanogel solution, a predetermined amount of dried nanogel was dispersed in PB solution.

### 3.6. Stability of Nanogels under Reductive Conditions

To investigate the stability and dissociation behavior of the nanogels by cleavage of the disulfide bonds between Dex and OLA chains under reductive conditions, PB solutions of GSH or DTT (10 mM) as reductive agents were added to the nanogel PB solutions (1 mg/mL). After certain periods, observation by the naked eye or hydrodynamic diameter measurements by DLS were carried out. Additionally, PB solutions of nanogels entrapping pyrene were prepared, and the fluorescence spectra were monitored after the addition of reductive agents.

### 3.7. Cell Viability Test

Mouse fibroblast NCTC clone 929 (L929) cells were obtained from the Health Science Research Resources Bank (HSRRB, Japan). Cell viability tests of nanogels were performed using the WST-8 assay kit against L929 cells. L929 cells (100 μL, 1 × 10^4^ cells/mL) were grown on 96-well microplates for 24 h in E-MEM containing 10% fetal bovine serum (FBS) and under a humidified atmosphere of 5% (*v*/*v*) CO_2_ at 37 °C. The cells were exposed to various concentrations (2.0 × 10^−4^ to 1.0 × 10^−1^ mg/mL) of nanogels and control samples dissolved in phosphate-buffered saline (−) [PBS(−)] for 23 h. Thereafter, 10 µL of WST-8 reagent was added to all cells and the cells were incubated for an additional 1 h (total 24 h). At the end of the incubation period, the microplates were read at 450 nm in a microplate reader. The control and background were chosen at a total of 110 μL of FBS-containing E-MEM with or without cells, respectively. The average background absorbance from the background wells was subtracted from the sample data. The values for each sample were in the linear region of the standard curve of the WST-8 assay. Data are expressed as the mean and SE (*n* = 6). The cell viability was calculated using the following equation:

cell viability (%) = (Nt/Nc) × 100,

where Nt and Nc are the numbers of cells with and without samples after 24 h of incubation, respectively. Sodium dodecyl sulfate (SDS, surfactant) was used as a positive control.

### 3.8. Cellular Uptake of Nanogels into HepG2 Cells

Cellular uptake of nanogels into human hepatocarcinoma (HepG2) cells having galactose receptors was investigated by flow cytometric analysis and confocal laser scanning microscopy (CLSM) using FITC-labeled nanogels. The introduction of FITC into Dex was carried out by mixing FITC with Dex. Dex (4.3 g, 100 μmol) and FITC (39 mg, 100 μmol) were dissolved in anhydrous DMSO (20 mL) under an Ar atmosphere and stirred overnight. Acetone was added to the reaction mixture to produce a precipitate. The obtained precipitate was dissolved in water and the aqueous solution was freeze-dried to give a yellow powder of FITC-Dex (3.85 g, yield 89%). FITC-labeled Dex-*g*-SS-OLA was prepared using FITC-Dex by a similar method as described above. The FITC-labeled EI_4_/Gal-Dex-*g*-SS-OLA nanogels were prepared by a dialysis method using a mixture of EI_4_/Gal-Dex-*g*-SS-OLA and FITC-labeled Dex-*g*-SS-OLA (9:1). Other FITC-labeled nanogels (Gal-Dex-*g*-SS-OLA, EI_4_-Dex-*g*-SS-OLA, EI_4_/Gal-Dex-*g*-OLA) were prepared by the same method.

The HepG2 cell line was purchased from RIKEN Cell Bank (RCB, Wako, Japan). HepG2 cells were cultivated in D-MEM containing 10% FBS in a humidified atmosphere of 5% (*v*/*v*) CO_2_ at 37 °C. Upon observation of cellular uptake of nanogels by flow cytometric analysis, the cells were seeded on 6-well culture dishes at 2.0 × 10^5^ cells/well and incubated for 24 h. FITC-labeled nanogels (200 µL, 1 mg/mL) dissolved in PBS(−) were added to the cell culture medium and incubated for 4 h at 37 °C. Cells were washed with PBS(−), treated by trypsin/ethylenediaminetetraacetic acid (EDTA) PBS(−) solution, and washed another three times with PBS(−). Cells were suspended in PBS(−) and counted by using a flow cytometer (Gallios, Beckman Coulter, Inc., Brea, CA, USA) with an argon-ion coherent beam laser (488 nm).

For the direct observation of cellular uptake of nanogels into the cells and their localization in each cell, the HepG2 cells were seeded on 35 mm culture dishes at 1 × 10^5^ cells and incubated for 24 h. Lysosomes of the cells were stained by LysoTracker RED for 40 min at 37 °C. The cells were washed three times with PBS(−), and the medium was replaced with fresh medium. FITC-labeled nanogels (1 mg/mL) dissolved in PBS(−) were added to the culture medium of the HepG2 cells and incubated for 5 min and a further 5 h at 37 °C. The cells were washed three times with PBS(−) and observed on a confocal laser scanning microscope (CLSM) (LSM5Pascal, Carl Zeiss Co., Ltd., Jena, Germany) at the predetermined incubation times.

## 4. Conclusions

We prepared nanogels composed of Dex with OLA chains attached via disulfide bonds, galactose residues, and oligo-amine groups to accelerate collapse under reductive conditions, facilitate cellular uptake by receptor-mediated endocytosis, and escape from endosomes by the proton sponge effect. The average hydrodynamic diameter of the EI_4_/Gal-Dex-*g*-SS-OLA nanogels was 285 nm, which decreased under reductive conditions. EI_4_/Gal-Dex-*g*-SS-OLA nanogels had no cytotoxicity against L929 fibroblast cells. Uptake of EI_4_/Gal-Dex-*g*-SS-OLA nanogels into HepG2 cells was confirmed by flow cytometric analysis and CLSM observations. The CLSM observations also suggested that EI_4_/Gal-Dex-*g*-SS-OLA nanogels uptaken by receptor-medicated endocytosis could escape from the endosomes and subsequently collapse in the cytosol. Although we did not incorporate drugs into the nanogels in this study, EI_4_/ligand-Dex-*g*-SS-OLA nanogels entrapping drugs can be expected to release the drugs efficiently to the cytosol during the collapse under reductive conditions, after ligand-receptor mediated endocytosis and endosomal escape. The biodegradable nanogels developed here, to control cellular traffic, should have great potential as drug delivery carriers for both hydrophobic and hydrophilic drugs.

## Supplementary Materials

Supplementary materials can be found at http://www.mdpi.com/1422-0067/19/6/1606/s1.
