# Supplementary material for: Preparation of Biodegradable Oligo(lactide)s-Grafted Dextran Nanogels for Efficient Drug Delivery by Controlling Intracellular Traffic
Source: Int J Mol Sci. 2018 May 30;19(6):1606. doi: 10.3390/ijms19061606 (PMC6032273; doi:10.3390/ijms19061606)
Supplement: Supplementary file 1 [file ijms-19-01606-s001.pdf]

Supporting Information

Preparation of Biodegradable Oligo(lactide)s-grafted Dextran Nanogels for Efficient Drug Delivery by Controlling Intracellular Traffic

Yuichi Ohya* [1, 2], Akihiro Takahashi,[2] Akinori Kuzuya,[1, 2]

[1] Department of Chemistry and Materials Engineering, Faculty of Chemistry, Materials and Bioengineering, Kansai University, 3-3-35 Yamate, Suita, Osaka 564-8680, Japan.

[2] Organizatin for Research and Development of Innovative Science and Technology (ORDIST), Kansai University, 3-3-35 Yamate, Suita, Osaka 564-8680, Japan.

*To whom correspondence should be addressed. yohya@kansai-u.ac.jp

Figure S1. ^{1}H NMR spectra of a) activated OLA (CI-OLA), b) Boc-cystamine-OLA and c) OLA-SS-NH_2 in $CDCl_3$.

Figure S2. ^{1}H NMR spectrum for hydrolysis products of EI_4/Gal-Dex-*g*-SS-OLA in NaOD/D_2O. The alkyl group at the terminal of OLA was not observed because of insolubility in aqueous solution.

Figure S3. Size distributions of the Dex-*g*-OLA, Dex-*g*-SS-OLA and EI_4/Gal-Dex-*g*-SS-OLA nanogels in PB solution measured by DLS.

Figure S4. Plots of fluorescence intensity ratio for I_1 (373 nm) to I_3 (383 nm) peaks of pyrene as a function of Dex-*g*-SS-OLLA concentration in PB solution.

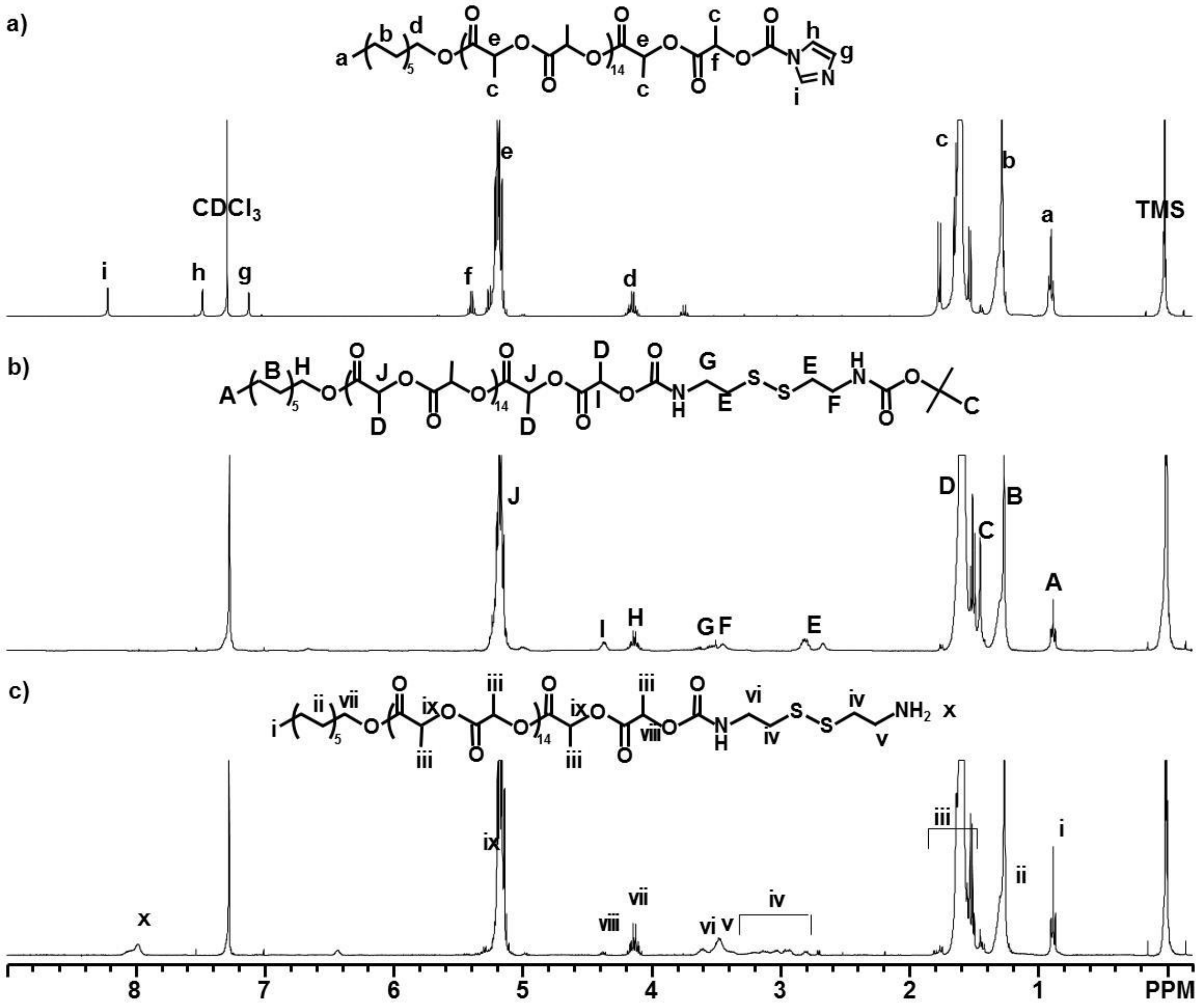

Figure S1. ^{1}H NMR spectra of a) activated OLA (CI-OLA), b) Boc-cystamine-OLA and c) OLA-SS-NH_2 in $CDCl_3$.

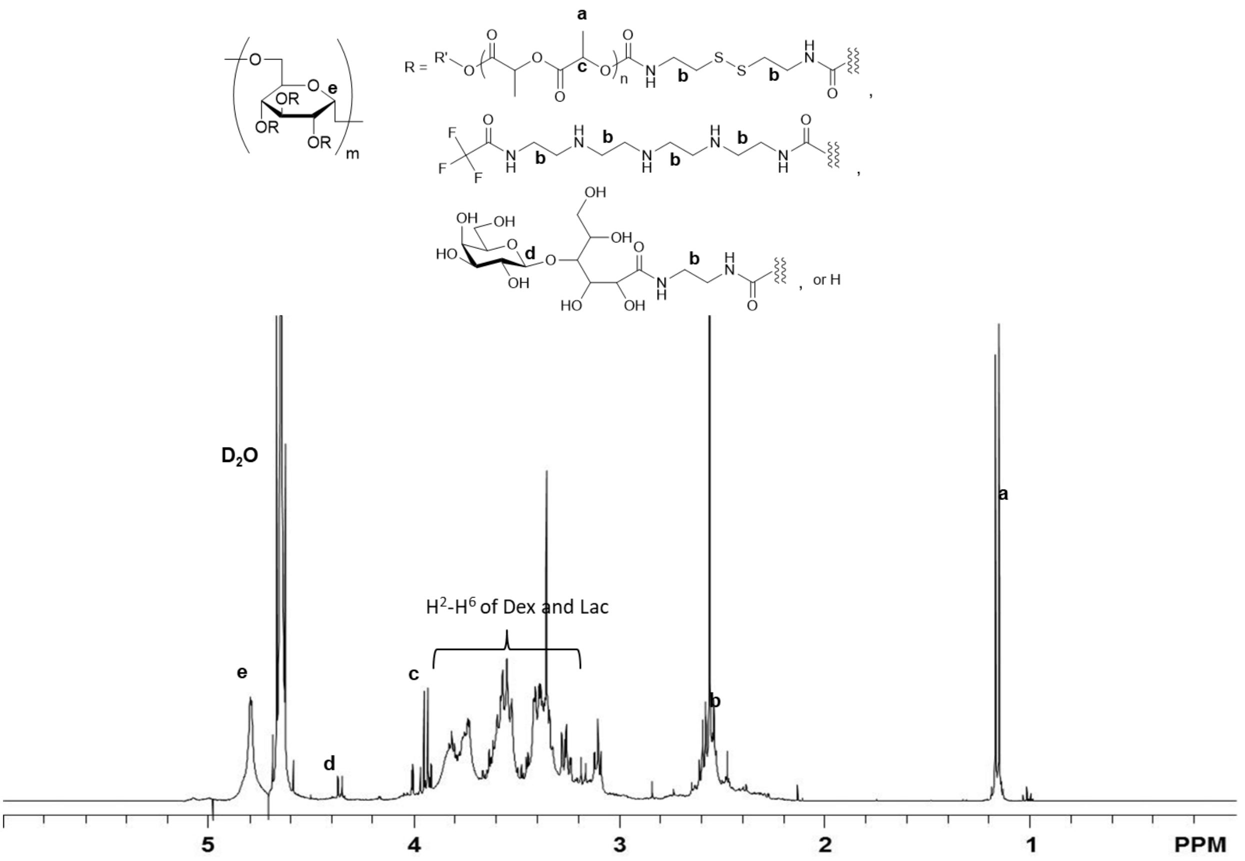

Figure S2. ^{1}H NMR spectrum for hydrolysis products of EI$_4$/Gal-Dex-*g*-SS-OLA in NaOD/D$_2$O.

The alkyl group at the terminal of OLA was not observed because of insolubility in aqueous solution.

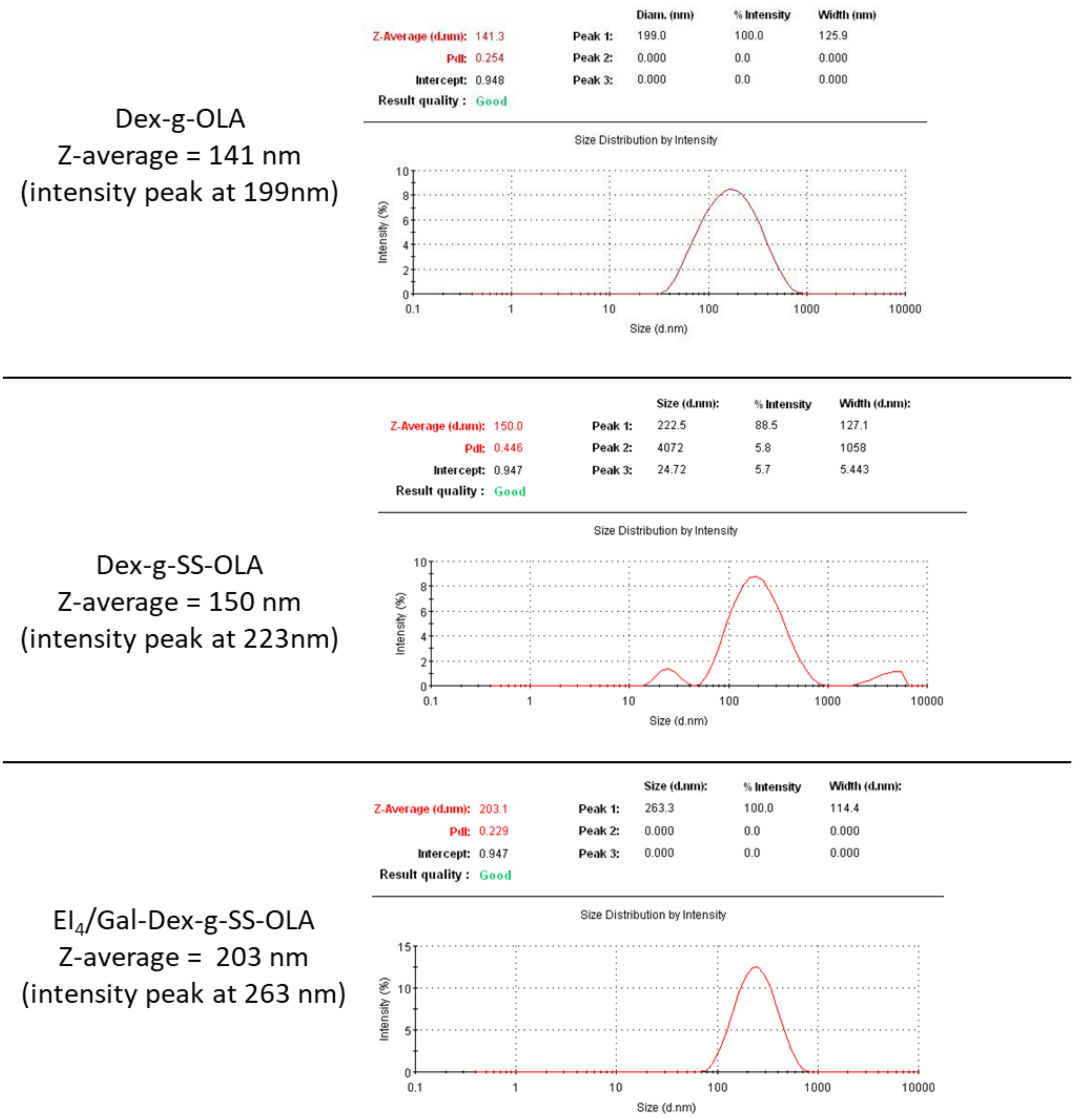

Figure S3. Size distributions of the Dex-*g*-OLA, Dex-*g*-SS-OLA and EI_4/Gal-Dex-*g*-SS-OLA nanogels in PB solution measured by DLS.

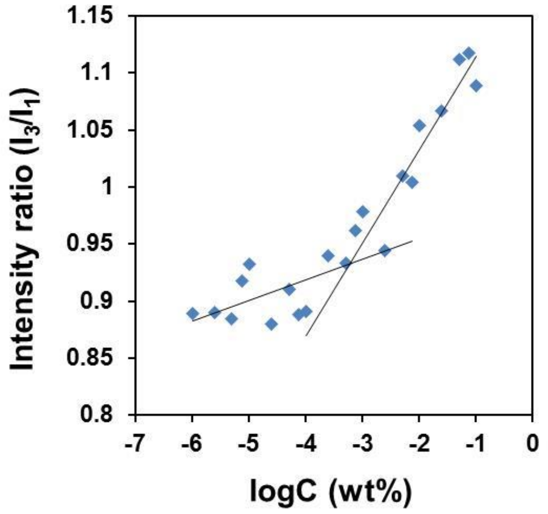

Figure S4. Plots of fluorescence intensity ratio for I_1 (373 nm) to I_3 (383 nm) peaks of pyrene as a function of Dex-*g*-SS-OLLA concentration in PB solution.
